# Structural connections in the brain in relation to gender identity and sexual orientation

**DOI:** 10.1038/s41598-017-17352-8

**Published:** 2017-12-20

**Authors:** Sarah M. Burke, Amir H. Manzouri, Ivanka Savic

**Affiliations:** 1 Brain & Development Research Centre, Department of Developmental and Educational Psychology, Leiden University, Leiden, The Netherlands; 2 Department of Women’s and Children’s Health, Karolinska Institute and University Hospital, Stockholm, Sweden; 3 Stressmotagningen, S:t Göransgatan 84, 112 38 Stockholm, Sweden

## Abstract

Both transgenderism and homosexuality are facets of human biology, believed to derive from different sexual differentiation of the brain. The two phenomena are, however, fundamentally unalike, despite an increased prevalence of homosexuality among transgender populations. Transgenderism is associated with strong feelings of incongruence between one’s physical sex and experienced gender, not reported in homosexual persons. The present study searches to find neural correlates for the respective conditions, using fractional anisotropy (FA) as a measure of white matter connections that has consistently shown sex differences. We compared FA in 40 transgender men (female birth-assigned sex) and 27 transgender women (male birth-assigned sex), with both homosexual (29 male, 30 female) and heterosexual (40 male, 40 female) cisgender controls. Previously reported sex differences in FA were reproduced in cis-heterosexual groups, but were not found among the cis-homosexual groups. After controlling for sexual orientation, the transgender groups showed *sex-typical* FA-values. The only exception was the *right inferior fronto-occipital tract*, connecting parietal and frontal brain areas that mediate own body perception. Our findings suggest that the neuroanatomical signature of transgenderism is related to brain areas processing the perception of self and body ownership, whereas homosexuality seems to be associated with less cerebral sexual differentiation.

## Introduction

Gender identity and sexual orientation belong to the most interesting facets of human biology. Yet, their underlying mechanisms are still unrevealed. Sexual orientation signifies the sex of the object of one’s sexual attraction, whereas gender identity denotes the sex and gender role one identifies with. A distinction between these two entities is particularly important when trying to understand the biological underpinnings of gender dysphoria (GD, DSM-5^1^), and its most common form, transgenderism, or transsexualism in ICD 10^2^. This might be complicated as there is higher prevalence of bi- and homosexuality among transgender compared to cisgender (cis- denotes not trans-) populations^3–7^. The signature of GD is cross-gender identification, discomfort with the own body, and feeling of estrangement to one’s physical sex. Both GD and sexual orientation are believed to be linked to prenatal and early post-natal sex hormone exposure^8–11^ leading to a less prominent sexual differentiation of the brain than in cisgender heterosexual individuals^12–15^.

More recent data partly challenge this model and propose that a neurobiological hallmark for GD is a fronto-occipito-parietal disconnection between neuronal circuits processing the perception of self and those mediating perception of body ownership^16–19^. While compelling, these data do not account for, nor explain the higher prevalence of bi- and homosexuality among transgender populations^3–7^. Most previous studies provide no information about sexual orientation or included heterosexual control groups [exceptions are^20^, who included controls with mixed sexual orientation, and^21^ including only non-homosexual transgender individuals)]. They thus risked to inevitably confound gender identity and sexual orientation. There are strong reasons to specifically investigate whether and how the neurobiology of GD incorporates interaction between gender identity and sexual orientation. Despite the rapid increase of neuroimaging studies in GD describing cortical thickness^16,22,23^, grey matter volume^24–26^, and structural connectivity^20,27–30^ this aspect has not been addressed.

As a first step in such an effort we report investigations with diffusion tensor imaging (DTI). With this MRI method, indices of white matter microstructure can be derived from the diffusion tensor, such as fractional anisotropy (FA), mean diffusivity, axial diffusivity, and radial diffusivity. For FA, a measure for the relative restriction of water diffusion along the axon, prominent sex differences have been reported, with higher FA values (indexing more axonal organization, fibre coherence, and myelination^31^), in several long white matter tracts, primarily the superior and inferior longitudinal fasciculus (SLF/ILF), cortico-spinal tract (CST), corpus callosum (CC), inferior fronto-occipital fasciculus (IFOF), and forceps minor, in men compared to women^28,32–39^.

We investigated FA values in 40 transgender men (TrM, female sex assigned at birth, mean age 24.0 ± 5.6 years) and 27 transgender women (TrW, male sex assigned at birth, 25.5 ± 5.4 years), together with 29 cisgender homosexual men (HoM, 30.6 ± 5.6 years), 30 cisgender homosexual women (HoW, 28.0 ± 6.1 years), as well as 40 cisgender heterosexual men (HeM, 29.4 ± 5.9 years) and 40 cisgender heterosexual women (HeW, 29.3 ± 5.5 years).

Several previous reports showed that FA values are greater in cisgender men than cisgender women in the SLF, ILF, CST, and CC. Given previous observations of a sex-reversed pattern of chemo-signal activation, amygdala connectivity, and hemispheric asymmetry in cisgender homosexual compared to cisgender heterosexual men and women^40–43^, we hypothesized that among HoM and HoW there could be signs of less pronounced masculinization/feminization, or even a sex reversed pattern of FA, than among our HeM and HeW. Diffusion values of TrM and TrW were previously reported to be in between those of cisgender groups^20,28,29^. However, based on our recent data^16–19^, we hypothesized that this could be a mere effect of the higher proportion of homosexuality among the transgender persons. Thus, we tested whether white matter sexual differentiation in the cisgender homosexual and transgender groups might be less pronounced or even sex-reversed by referring to their FA values as being sex-(a)typical, i.e. (not) in correspondence with their assigned sex at birth.

A further hypothesis was that transgender individuals, independently of their sexual orientation, would differ from cisgender individuals with respect to fronto-parieto-occipital connections involved in own body perception in the context of self, and, specifically, in the IFOF, a tract that connects these regions^44^. If true, this could be a potential neural correlate of GD.

## Results

### Sample characteristics

The six groups (40 TrM, 27 TrW, 29 HoM, 30 HoW, 40 HeM, and 40 HeW) differed significantly in terms of mean age, *F*(5, 200) = 7.0, *p* < 0.001 (Table 1). Therefore, age was added as covariate of no interest to all further analyses.

**Table 1:** Subject characteristics.

	HeM				HoM				**TrW**				*F*(df)	*p*-value
	N	mean	SD	range	N	mean	SD	range	N	mean	SD	range		
Age in years	40	29.4	5.9	20–42	29	30.6	5.6	22–45	27	25.5	5.4	19–40	6.3(2,93)	**0.003**
Sexual orientation^a^	39	0.3	0.5	0–2	29	5.7	0.5	5–6	25	3.5	0.2	0–6	163.4(2,90)	**<0.001**
ICV in mL	40	1632.1	130.7	1331.0–1887.6	29	1618.3	123.1	1419.1–1890.0	27	1622.5	123.7	1427.0–1864.9	0.1(2,96)	0.896
	**HeW**				**HoW**				**TrM**					
	**N**	**mean**	**SD**	**range**	**N**	**mean**	**SD**	**range**	**N**	**mean**	**SD**	**range**	***F*** **(df)**	***p*** **-value**
Age in years	40	29.3	5.5	20–43	30	28.0	6.1	19–40	40	24.0	5.6	18–42	9.1(2,107)	**<0.001**
Sexual orientation^a^	40	0.4	0.6	0–2	30	5.5	0.6	4–6	37	4.4	1.7	0–6	207.0(2,104)	**<0.001**
ICV in mL	40	1426.0	117.1	1044.4–1747.6	30	1420.2	130.9	1075.8–1671.3	40	1403.1	141.8	1014.0–1696.7	0.3(2,110)	0.719

^a^Sexual orientation was assessed using the self-report Kinsey scale^57^, scores range from 0 = “exclusively heterosexual” to 6 = “exclusively homosexual” in relation to one’s birth-assigned sex; ICV = total intra-cranial volume; HeM = heterosexual cisgender men; HoM = homosexual cisgender men; TrW = transgender women; HeW = heterosexual cisgender women; HoW = homosexual cisgender women; TrM = transgender men.

By design, the groups differed in terms of sexual orientation. In contrast to the cisgender groups, the transgender groups were heterogeneous with regard to sexual orientation. Out of 40 included TrM 24 (60%) identified as gynephilic (scores 4–6), eight (20%) as bisexual (score 3), and five (12.5%) as androphilic (scores 0–2). Out of 27 included TrW 15 (55.5%) identified as androphilic, (scores 4–6), nine (33.3%) as gynephilic (scores 0–2), and one (3.7%) as bisexual (score 3). Self-report Kinsey scores of three TrM, and two TrW were missing.

ICV were not significantly different between groups of the same assigned sex at birth (males: *F*(2,96) = 0.1, *p* = 0.896; females: *F*(2,110) = 0.3, *p* = 0.719), see Table 1. Thus, ICV in TrM and TrW followed their sex assigned at birth, and were significantly larger in cisgender men and TrW, than in cisgender women and TrM.

### Voxel-wise whole brain analysis

**Figure 1:**
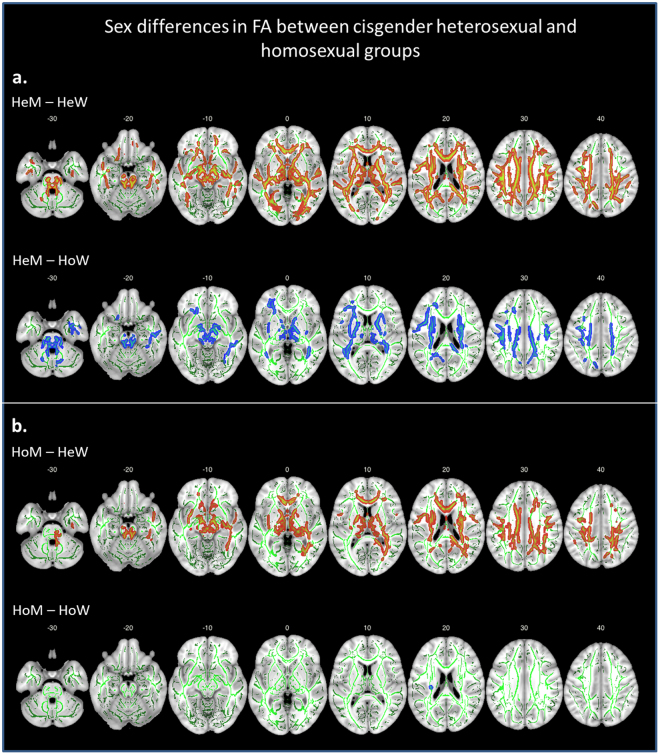
Whole brain voxel-wise sex differences in FA (*p* < 0.05, *FWE*-corrected) between cisgender homosexual and heterosexual groups, projected on the mean FA skeleton (green) using the “tbss fill” procedure in FSL. Part a. shows the comparisons between heterosexual men (HeM) and heterosexual women (HeW, red), and homosexual women (HoW, blue). Part b. shows the comparisons between homosexual men (HoM) and HeW (red) and HoW (blue). Slice labels indicate MNI z-coordinates. L = left, R = right.

A Sex (male, female) by Sexual orientation (heterosexual, homosexual) ANOVA including the four cisgender groups revealed a significant (*p* < 0.05, Family Wise Error (FWE)-corrected) main effect of Sex, with men showing higher FA values than women in several white matter tracts, with the largest clusters located in the bilateral CST and left SLF (Table 2). To the contrary, there was no main effect or interaction with Sexual orientation. Post-hoc two-group comparisons revealed that FA values in HeM were significantly higher than in HeW along the long tracts, the CST, SLF, ILF, CC (Fig. 1a). HeM had significantly higher FA values also compared to HoW, although differences were less extensive than in relation to HeW (Fig. 1a).

**Table 2:** Results of a whole-brain, voxel-wise 2 (Sex) by 2 (Sexual orientation) ANOVA, including all four cisgender groups; results are displayed at *p*_*FWE*_ < 0.05, cluster threshold of *t* = 2.3, and a minimal cluster size of *k* ≥ 100 voxel (1 mm^3^ voxel size); CST = cortico-spinal tract; SLF = superior longitudinal fasciculus; WM = white matter; IFOF = inferior fronto-occipital fasciculus; ILF = inferior longitudinal fasciculus; L = left; R = right.

Region	cluster size, *k*	max. F-value	MNI-coordinates		
			x	y	z
*Main effect Sex*					
R CST	9931	38.7	12	−20	−1
L CST	3581	36.9	−20	−5	18
L SLF	1808	19.1	−42	−8	25
L cerebellar WM	900	32.9	−19	−60	−34
R cerebellar WM	841	28.3	43	−53	−33
L forceps minor	811	22.4	−12	30	−2
R forceps minor	636	17.8	22	29	30
R forceps minor	635	16.5	8	30	4
R IFOF (orbito-frontal)	245	12.7	33	31	−5
L ILF	230	16.2	−39	−19	−22
R ILF	222	12.8	37	−46	−12
R ILF	209	19.0	39	−25	−21
L ILF	201	15.4	−48	−3	−13
R SLF, temporal part	192	13.0	42	−58	−4
R CST	179	18.0	25	1	45
R ILF	176	20.0	50	−11	−22
R posterior cingulum	149	21.5	11	−41	31
L ILF	145	16.3	−52	−13	0
R SLF	140	15.2	49	0	18
R uncinate fasciculus	136	13.8	18	26	−14
R ILF	135	14.8	33	1	−30
R SLF	116	11.0	36	−60	25
L SLF, temporal part	114	24.2	−46	−39	−5
L SLF	106	19.3	−44	−31	25

Like HeM, but less pervasive, HoM had higher FA values than HeW in several bilateral tracts (Fig. 1b). Notably, the comparison between HoM and HoW revealed only a small cluster located in the left CST where HoM had significantly higher FA (Fig. 1b). In order to rule out the possibility that the clear sex difference between HeM and HeW, compared to the rather small differences between HoM and HoW was due to the smaller sample sizes of the homosexual groups, we conducted an additional tbss analysis between smaller, randomly selected sub-samples of HeM (*N* = 30) and HeW (*N* = 30). This analysis replicated our previous finding in larger groups and showed significantly higher FA values in HeM than HeW in several bilateral tracts (see Supplementary Table S1). Thus, in contrast to the highly significant sex difference between heterosexual men and women, the homosexual groups differed barely from each other.

There were no tracts with higher FA values in cisgender women than men, irrespective of sexual orientation. FA values in HoM did not differ from those in HeM, and there were no differences between HoW and HeW.

Next, we tested whether the transgender groups would show sex-atypical FA values. A Sex (male/female sex assigned at birth) by Gender identity (male, female) ANOVA was carried out including all cisgender participants (HeM, HoM, HeW, HoW) and the two transgender groups (TrW, TrM). We found no significant (*p*_*FWE*_ ≤ 0.05) interaction, no main effect of Gender identity in any of the tracts tested, but again a main effect of Sex with the largest clusters of higher FA located in the right IFOF (*k* = 5802, *F* = 30.8), left thalamic radiation (*k* = 1435, *F* = 42.3), right splenium of the CC (*k* = 958, *F* = 31.3), in participants with a male sex assigned at birth than in those with a female sex assigned at birth (see Supplementary Table S2).

**Figure 2:**
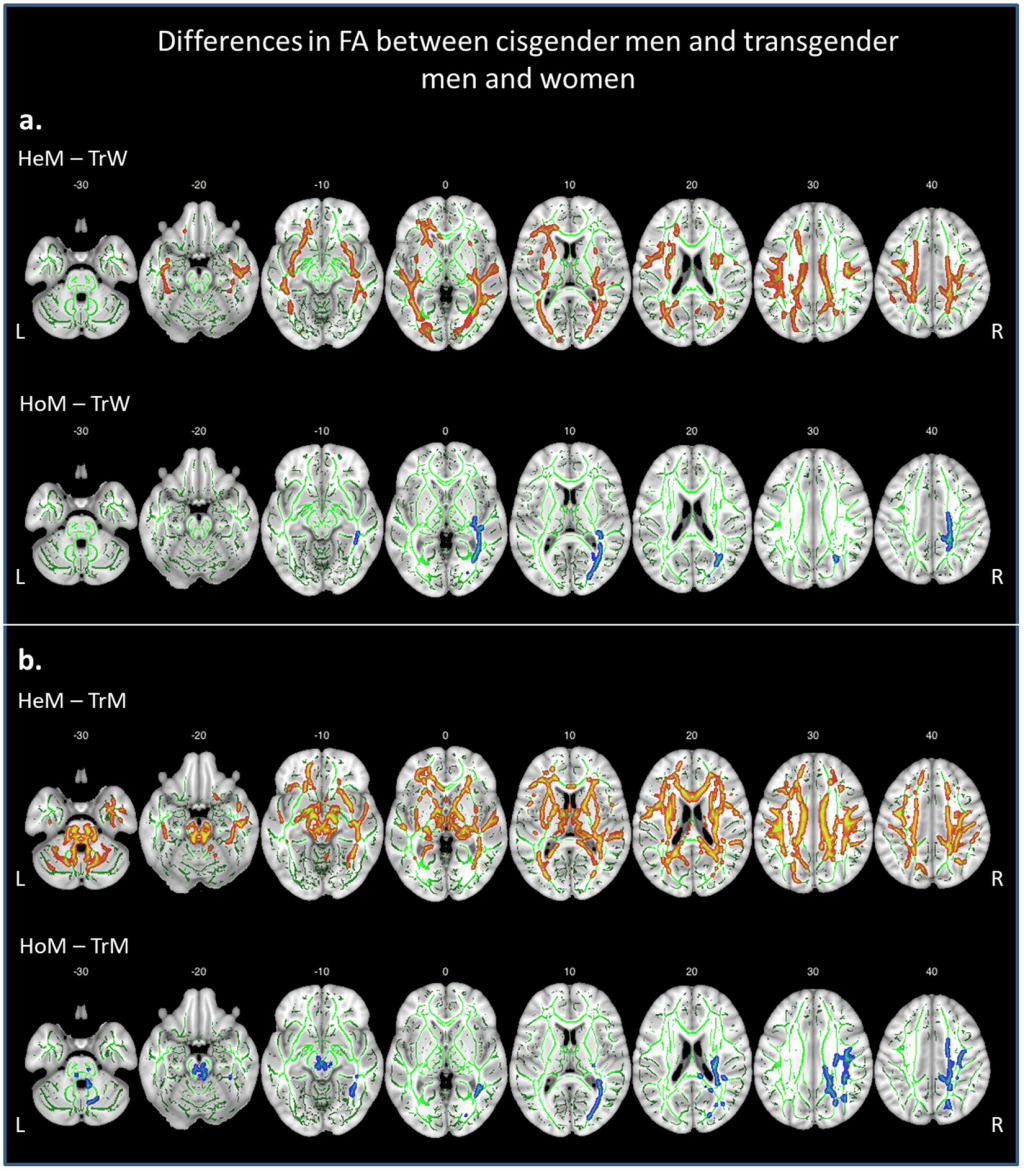
Whole brain voxel-wise group differences in FA (*p* < 0.05, *FWE*-corrected) between homo- and heterosexual cisgender men (HoM/HeM) and transgender women (TrW, part a), and transgender men (TrM, part b). The lower panel of part b. HoM – TrM, is displayed at a slightly more lenient threshold of *p* < 0.06, *FWE*-corrected. Significant clusters are projected on the mean FA skeleton (green) using the “tbss fill” procedure in FSL; Slice labels indicate MNI z-coordinates; L = left, R = right.

Separate comparisons between the three groups with male sex assigned at birth, revealed lower FA values in **TrW** than in HeM in the bilateral SLF (left: *k* = 7771, *p*_*FWE*_ = 0.015; right: *k* = 124, *p*_*FWE*_ = 0.042), bilateral IFOF (left: *k* = 4709, *p*_*FWE*_ = 0.022; right: *k* = 5516, *p*_*FWE*_ = 0.018), and right CST (*k* = 3298, *p*_*FWE*_ = 0.013) (Fig. 2a, Table 3a). Notably, and not tested earlier, the difference between TrW and HoM was much less extensive, restricted to the right IFOF (*k* = 1030, *p*_*FWE*_ = 0.027) and CST (*k* = 448, *p*_*FWE*_ = 0.039) (Fig. 2a, Table 3a). The corresponding comparisons between the three groups with female sex revealed no significant differences in FA.

**Table 3:** Table 3 corresponds with Fig. 2. Part (**a**) shows post-hoc comparisons of a one-way ANOVA (with age as covariate) including heterosexual and homosexual cisgender men (HeM, HoM), and transgender women (**TrW**); results are displayed at *p*_*FWE*_ < 0.05, and a minimal cluster size of *k* ≥ 100 voxel (1 mm^3^ voxel size); Part (**b**) shows the resulting clusters of a *t*-test between HeM and transgender men (TrM) displayed at *p*_*FWE*_ < 0.05, cluster threshold of *t* = 2.3, and minimal cluster size of *k* ≥ 100 voxel; CST = cortico-spinal tract; SLF = superior longitudinal fasciculus; IFOF = inferior fronto-occipital fasciculus; L = left; R = right.

(**a**)	cluster size, *k*	*p*_*FWE*_-*value*	MNI-coordinates		
Region			x	y	z
*HeM* - *TrW*					
L SLF	7771	0.015	−18	−7	42
R IFOF, posterior part	5516	0.018	38	−47	−7
L IFOF, posterior part	4709	0.022	−28	−64	18
R CST	3298	0.013	20	−23	39
R SLF	124	0.042	33	−33	36
*HoM* - *TrW*					
R IFOF, posterior part	1030	0.027	33	−65	9
R CST	448	0.039	19	−39	39
R IFOF	288	0.042	37	−35	7
(**b**)	**cluster size**, ***k***	**max**. ***t***-**value**	**MNI**-**coordinates**		
Region			**x**	**y**	**z**
*HeM* - *TrM*					
R CST	1065	6.6	3	−38	−30
R SLF	751	5.0	14	−15	31
R SLF	260	4.6	39	−8	30
L anterior thalamic radiation	188	4.3	−4	−33	−25
L CST	155	4.8	−28	−22	22
L CST	147	4.5	−9	−24	−29
L CST	125	4.2	−19	−13	44
L SLF	112	3.9	−46	−7	23

Independent *t*-tests, investigating sex differences in FA, showed that TrW did not differ from HoW or HeW, and thus *showed sex-atypical FA values*. In contrast, **TrM** had lower FA than HeM, but this difference was less extensive than the differences in FA between HeM and the cisgender females, and restricted to the bilateral CST (left: *k* = 155, *t* = 4.8; *k* = 147, *t* = 4.5; *k* = 125, *t* = 4.2; right: *k* = 1065, *t* = 6.6) and SLF (left: *k* = 112, *t* = 3.9; right: *k* = 751, *t* = 5.0; *k* = 260, *t* = 4.6) (Fig. 2b, Table 3b), but *not IFOF*. Notably, there was no significant difference between TrM and HoM (*p*_*FWE*_ < 0.05). Using a slightly more lenient threshold of *p*_*FWE*_ < 0.06 revealed, however, a higher FA in HoM, but only in the right CST (*k* = 89, *t* = 4.7), right SLF (*k* = 83, *t* = 5.6; *k* = 72, *t* = 3.7), and right IFOF (*k* = 67, *t* = 3.6; *k* = 22, *t* = 3.8; (*k* = 21, *t* = 2.8), (Fig. 2b). A direct comparison of the two transgender groups showed no significant differences in FA.

Thus, in the whole-brain voxel-wise analyses, there was a general effect of Sex, but not Sexual orientation. The results indicated a gradual decrease of FA across the 4 cisgender groups, such that HeM > HoM > HoW > HeW. In addition, in both transgender groups we observed more pronounced and bilateral differences in FA in relation to HeM, whereas compared to HoM lower FA in TrM, just as in TrW, was less pervasive and restricted to right hemispheric tracts such as the IFOF, CST, and SLF.

### Tract-wise mean FA analyses

In order to more specifically differentiate between the contribution of sexual orientation and having a cross-gender identity to less pronounced sexual differentiation of white matter microstructure, we extracted mean FA values for tracts found to be sex dimorphic. Similar as with the whole-brain analyses, we carried out Sex by Sexual orientation ANOVA, Sex by Gender identity ANOVA, and one-way ANOVA followed by post-hoc tests. FA value comparisons including the transgender groups were done both with and without (see Supplementary Information) scores on the Kinsey scale as covariate, in order to account for their more variable sexual orientation.

### FA and Sexual orientation

**Figure 3:**
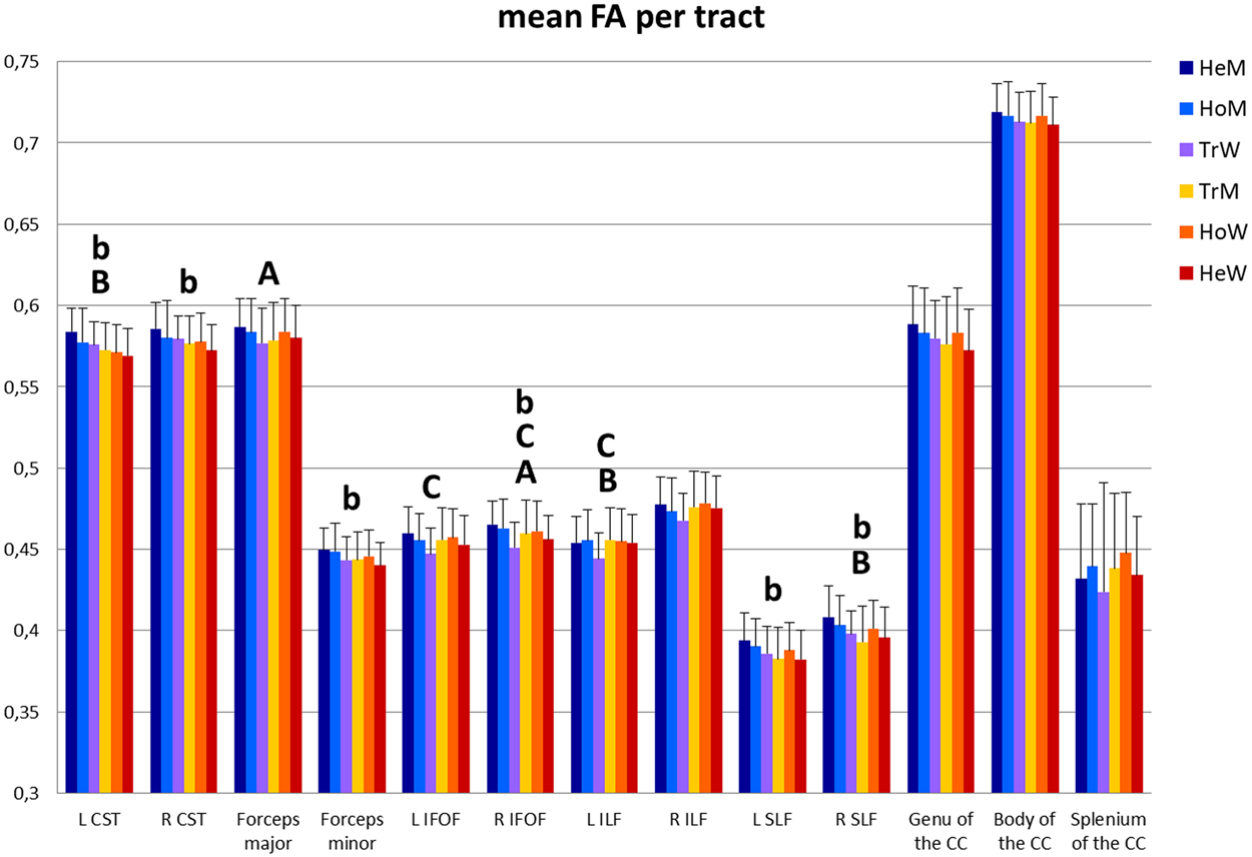
Group differences in mean (±SD) FA per tract were analysed with a Sex (male/female sex assigned at birth) by Gender identity (male/female) multivariate ANOVA including all cisgender and both transgender groups, with age and Kinsey scores included as covariates. Upper case letters denote significant (*p* < 0.05) (A) interaction effects, (B) main effects of Sex, and (C) main effects of Gender identity. Also, results of a Sex (male/female sex assigned at birth) by Sexual orientation (homo-/heterosexual) multivariate ANOVA including the four cisgender groups, with age as covariate, are displayed. Lower case letters denote significant (a) interaction effects, (b) main effects of Sex, and (c) main effects of Sexual orientation. HeM = heterosexual cisgender men, HoM = homosexual cisgender men, TrW = transgender women, TrM = transgender men, HoW = homosexual cisgender women, HeW = heterosexual cisgender women, CST = cortico-spinal tract, IFOF = inferior fronto-occipital fasciculus, ILF = inferior longitudinal fasciculus, SLF = superior longitudinal fasciculus, CC = corpus callosum, L = left, R = right.

A 2 (Sex) by 2 (Sexual orientation) multivariate ANOVA including the four cisgender groups revealed a significant main effect of Sex, with men showing higher FA than women in the bilateral CST (left: *p* < 0.001; right: *p* = 0.010), forceps minor (*p* = 0.010), bilateral SLF (left: *p* = 0.023; right: *p* = 0.021), right IFOF (*p* = 0.036), and a trend in the genu of the CC (*p* = 0.058) (Fig. 3). There was no main effect of Sexual orientation, and no significant interaction.

One-way ANOVA between the four cisgender groups revealed significant differences in the bilateral CST (left: *p* = 0.001; right: *p* = 0.013), forceps minor (*p* = 0.022), bilateral SLF (left: *p* = 0.029; right: *p* = 0.022), and genu of the CC (*p* = 0.022). Post-hoc two-group comparisons revealed significant sex differences in mean FA in all these tracts between the heterosexual groups [bilateral CST *p* < 0.001, forceps minor *p* < 0.003, bilateral SLF (left: *p* = 0.004; right: *p* = 0.003), genu of the CC *p* = 0.005], but, notably, like in the whole-brain comparisons, there were no differences between the homosexual groups in any of the tracts.

HoM had significantly higher FA than HeW in the left CST *(p* = 0.048), and forceps minor *(p* = 0.017), and showed trends in the right CST *p* = 0.058 and left SLF *p* = 0.055. But HoM had similar FA values as HeM (Fig. 3).

In comparison with HeM HoW showed significantly lower FA only in the left CST (*p* = 0.002) and HoW had seemingly, but not significantly higher FA values than HeW (Fig. 3).

Thus, the tract-specific analyses suggested that the non-significant sex differences in FA between the two homosexual groups were mainly explained by relatively increased FA values in HoW.

### FA and Gender identity - accounting for sexual orientation

A 2 (Sex) by 2 (Gender identity) multivariate ANOVA, adding Kinsey scores next to age as covariates of no interest revealed a significant interaction effect in the right IFOF (*p* = 0.014) and forceps major (*p* = 0.043). Notably, the main effect of Gender identity was significant only for the bilateral IFOF (right: *p* = 0.007; left: *p* = 0.033) and left ILF (*p* = 0.039), whereas the main effect of Sex was significant in the left CST (*p* = 0.009), right SLF (*p* = 0.020), left ILF (*p* = 0.045), and sub-significant in the right CST (*p* = 0.053).

One-way ANOVA among groups with the same sex assigned at birth, after accounting for individual differences in sexual orientation, showed significant differences among males only for the IFOF (right: *p* < 0.001; left: *p* = 0.018) and left ILF (*p* = 0.024). Here, HoM had higher FA values than TrW (right IFOF *p* = 0.002, left IFOF *p* = 0.008; left ILF *p* = 0.008), also HeM had higher FA than TrW, but significantly only in the right IFOF (*p* = 0.006).

Among the (at birth assigned) females, no differences were seen in mean FA of the IFOF, but a group difference appeared in the splenium of the CC (*p* = 0.008), with HeW showing significantly (*p* = 0.017) lower and HoW showing a trend (*p* = 0.082) for higher FA values than TrM. This result was driven by one TrM. After exclusion of this participant, the group difference in the splenium disappeared.

A direct comparison between TrW and TrM, using Kinsey scores as covariate, showed no significant group differences in mean FA in the IFOF, but in the left ILF TrM had significantly higher mean FA than TrW (*p* = 0.025).

Thus, accounting for individual differences in sexual orientation, the transgender groups showed lower, sex-atypical FA specifically in the right IFOF and left ILF. In all other tracts, FA values of the transgender groups *became sex-typical after accounting for sexual orientation* (see for comparison Supplementary Results when Kinsey scores were not co-varied).

As a second approach to investigate the contribution of transgender persons’ sexual orientation in explaining sexual differentiation of white matter microstructure, we conducted additional tract-wise mean FA analyses, differentiating between homosexual and non-homosexual (i.e. heterosexual or bisexual) transgender sub-groups.

The four at birth male assigned groups (14 homosexual TrW, 10 non-homosexual TrW, 27 HoM, 37 HeM) differed significantly only in the IFOF (right: *p* = 0.001; left: *p* = 0.016), and left ILF (*p* = 0.048). More specifically, for the left ILF HeM did not show significant differences between either homosexual (*p* = 0.068) or non-homosexual (*p* = 0.161) TrW, whereas HoM had significantly higher FA in the ILF compared to both homosexual (*p* = 0.010) and non-homosexual (*p* = 0.020) TrW. In the left IFOF, HeM showed significantly higher FA than homosexual TrW (*p* = 0.029), only sub-significantly higher FA than non-homosexual TrW (*p* = 0.060), whereas HoM had significantly higher mean FA compared with both non-homosexual TrW (*p* = 0.013), and homosexual TrW (*p* = 0.010). For the right IFOF, both cisgender male groups had significantly higher mean FA than homosexual TrW (comparison with HeM *p* = 0.001; with HoM *p* = 0.003), as well as non-homosexual TrW (comparison with HeM *p* = 0.005; with HoM *p* = 0.012).

Again, there were no tract-wise FA differences between the four at birth assigned female groups (24 homosexual TrM, 13 non-homosexual TrM, 27 HoW, 36 HeW). However, specifically in the IFOF and left ILF both homosexual and non-homosexual TrM also did *not* differ from the cisgender males, *thus, showing in-between sex FA values independently of sexual orientation*.

In summary, the group comparisons of mean FA in specific tracts, also after accounting for the mixed sexual orientation among the transgender groups, explicitly confirmed a special role for the right IFOF in transgenderism or GD, differentiating between FA values of cisgender men (HoM and HeM) and TrW, with non-homosexual TrW showing the lowest FA values across groups.

## Discussion

The present study investigates whether and how structural white matter connections, indexed by FA are related to gender identity and sexual orientation. Specifically, and based on several of our own recent studies^16–19^ we wondered whether different FA values in the IFOF compared to sex (assigned at birth)- matched controls can be regarded as a neural correlate of GD. The study also explored whether and how sexual orientation *per se* is related to FA in the long white matter tracts of the brain, consistently showing sex differences among cisgender heterosexual controls^28,32–39^. Several previous neuro-imaging studies have suggested that sexual differentiation of the brain is less pronounced in transgender individuals^12–15^, but none did specifically investigate sex, gender identity and sexual orientation in the same setting, directly comparing transgender groups with both cisgender homosexual and heterosexual controls. We found a significant main effect of *Sex* in several major white matter tracts (with higher FA in males), but, notably, not for *Sexual orientation*. Furthermore, congruent with our primary hypothesis there was a significant effect of *Gender identity* in the right IFOF. In the other tracts measured, the present study revealed, like in several previous studies, *sex-atypical* FA values in transgender individuals. However, and importantly, these values became *sex-typical* after accounting for sexual orientation.

At variance to the cis-heterosexual controls HoM and HoW displayed almost no differences in FA (Fig. 1b). Moreover, HoW had significantly lower mean FA compared with HeM only in the left CST, and relative to HeW HoW had seemingly, but non-significantly higher, and thus more male-typical FA values (Fig. 3). To the contrary, the direct comparison between HoM and HeM showed no significant differences. Together, these data argue for less pronounced sexual differentiation of these white matter tracts among homosexual compared to heterosexual cisgender persons, possibly more due to a masculinization of long tracts in HoW, and to a lesser degree a de-masculinization in HoM. In line with reports about testosterone promoting white matter growth^45^, such androgen effects are hypothesized to be linked to homosexuality^8–11^. Homosexual men were previously found to have a more female-like interstitial nucleus of the anterior hypothalamus^46^, thinner cuneus cortex, and smaller thalamic volumes compared with heterosexual men^47^. The finding of more pronounced sex-atypical characteristics in HoW than HoM is at odds to reports of a less consistent sex-atypical neuropsychological test performance in HoW^48,49^, and needs further evaluation. Male and female homosexuality may not be analogously manifested at a structural level in the human brain.

With regard to GD the major observation was that the significant effect of Gender identity, after accounting for sexual orientation, was confined to the IFOF. Since there were no significant differences in FA between TrM and cisgender women, sex-atypical FA in the IFOF might be a more prominent feature in TrW. Interestingly, both TrW and TrM displayed FA values in the CST and SLF more in accordance with their sex assigned at birth, whereas in the IFOF the FA values were more in accordance with their gender identity (sex-atypical for TrW, sex-in between for TrM). The IFOF connects the occipital, parietal and medial prefrontal cortices^44^. The IFOF, in particular on the right side, may thus be involved in the perception of self (mediated by the mPFC^50^) and the perception of one’s own body (mediated by the right parietal lobe^51,52^). Consequently, aberrant FA in the IFOF of transgender individuals may be underlying to the unconformity between their perception of self and their body. Importantly, this finding of sex-atypical FA values in the IFOF did not change after accounting for the more heterogeneous sexual orientation among the transgender participants. Moreover, in our recent longitudinal study on testosterone treatment effects in a sub-sample of the TrM described here, we observed increases of FA selectively in the posterior part of the right IFOF, which may indicate treatment-related improvement of white matter integrity^18^.

TrM overall showed a female-typical pattern with lower FA values than HeM, again, except for the IFOF. In contrast, compared to HoM TrM had similar mean FA values (Fig. 2b). Accordingly, in most tracts HoW had higher or similar FA values compared with TrM. Interestingly, Rametti *et al*.^28^, reported seemingly different results from ours with *significant* male-typical FA values in the right SLF and forceps minor in TrM. However, they investigated only 18 gynephilic (homosexual in relation to the sex assigned at birth) TrM. Our group of 40 TrM was thus larger and more heterogeneous in terms of sexual orientation. Although we did not find any significant differences among the (at birth assigned) female groups, considering the present observations, it is possible that, at least to some extent, the findings by Rametti *et al*.^28^ might be attributed to sexual orientation rather than gender identity.

This said, it is of note that in contrast to the cis-heterosexual groups, both the transgender samples and the cis-homosexual groups did not show the normative sex difference pattern with males (at birth assigned sex) having higher FA than females. Furthermore, whereas the differences in voxel-wise FA between the transgender groups and HeM were overall bilateral and more pervasive, their deviation from HoM was restricted to right hemispheric and smaller clusters. Together with the observed masculinization effects in HoW, and in line with the literature, this argues for less pronounced sexual differentiation of white matter in *both* homosexual cisgender individuals^8–11^, and in transgender persons^12–15^, compared with our cis-heterosexual groups, and in agreement with the notion of higher co-occurrence of transgenderism and homosexuality.

Importantly, the present data also provide a neuroanatomical underpinning for a GD/transgender-specific aspect - the body dysphoria and great distress due to incongruence between physical sex and experienced gender. The right-hemispheric differences between cis-homosexual and transgender groups, together with the confirmed aberration of FA in the (right) IFOF, provide compelling indications for the hypothesized different own body perception, specifically in transgender individuals. In line with our results, several previous neuroimaging studies found differences between trans- and cisgender groups particularly located in the right hemisphere^16,17,22,27,29^, more specifically in the right insula, (pre-) cuneus, temporo-parietal junction, orbito-frontal, medial frontal, and anterior cingulate cortex. These regions, and the right hemisphere in general have been reported to be involved in cognitive processes of (body) perception in relation to self, body ownership, ego-centric representation, and bodily self-consciousness^51–55^.

The crucial methodological aspects of the present study were the inclusion of both cis-heterosexual and cis-homosexual control groups, and the use of Kinsey scores as covariate. However, adding scores on the sexual orientation questionnaire as covariate might not be considered a valid statistical approach, because the assumption of independence between the independent variable, i.e. group, and the covariate is clearly violated in case of our four cisgender groups. Also, ANCOVA is generally used to account for small differences on the covariate, but in our model cisgender groups scoring on the two extremes of the Kinsey scale (homosexual versus heterosexual) were compared, and, moreover, this group difference was introduced by design. However, an argument in favour of conducting ANCOVA, including Kinsey scores as covariate is that we did not randomly assign subjects to the various groups, but considered group as a categorical predictor variable that is observed, and is not manipulated. Therefore, the independence assumption between the covariate and the independent variable (group) becomes irrelevant, and consequently our approach can be considered valid.

We binarized sexual orientation, including only those cisgender participants with Kinsey scale scores on the extreme ends (see inclusion criteria). One might argue that sexual orientation should rather be treated as continuous variable. However, because one of our specific aims was to address the issue of sexual orientation in relation to FA, this categorization was considered necessary as a first step to compare the extremes on the sexual orientation scale.

At variance to some more recent data by^20^, we do not report axial and radial diffusivity in this study. The underlying rationale is that non-FA diffusion measures are generally used to further characterize FA, but variations as a function of sex/gender have primarily been reported for FA. Radial and axial diffusivity will be elaborated on in a separate study.

Our groups differed in terms of mean age, which is suboptimal, because FA changes with aging. We accounted for these age differences by adding age as a covariate of no interest to all analyses.

We did not assess sex steroid hormone levels in our cisgender participants, but have no reason to assume that testosterone levels were higher in HoW than HeW, which might explain their relatively masculinized FA values, considering that no participant reported use of anabolic steroids, and none had any hormonal deficiency or aberration (see exclusion criteria).

In conclusion, the present findings support the idea of a distinction and partial overlap between the neurobiology underlying sexual orientation and transgenderism. Moreover, the observed right-hemisphere differences between the transgender groups and cisgender controls, also after taking into account sexual orientation, specifically in the IFOF further emphasize that the signature of GD is related to self-processing and the experience of body ownership.

## Materials and Methods

### Participants

The transgender participants were recruited by the Gender Team of the ANOVA Center of expertise in Andrology, Sexual Medicine, and Transgender Medicine, Karolinska University Hospital (Stockholm, Sweden), specialized for the evaluation and treatment of individuals with GD. All consecutively arriving adults aged 18–45 years who sought gender confirming medical interventions, and were diagnosed with transsexualism based on the ICD-10 diagnostic criteria^2^, were approached to enter the study between January 2011 and June 2016. Exclusion criteria consisted of previous or current hormonal treatment, any known chromosomal or hormonal disorder, any current psychiatric disorder [as confirmed by the Mini International Neuropsychiatric Interview (M.I.N.I.)^56^], any neurological or other major medical disorder, or any medications with psychotropic effects (antipsychotic or antiepileptic agents, lithium, benzodiazepines or opioid analgesics). Eligibility for participation was based on clinical interviews and available medical records after permission of the participant. We excluded individuals with known autism spectrum disorder (ASD) (diagnosed before being referred to the team) or participants who showed clinical signs of ASD when being assessed by the team. Cisgender controls were recruited via friends and advertisements at the Karolinska Institute campus, and through local “LGBTQ” organizations. Exclusion criteria for the control groups included GD, neurological or psychiatric disorders, substance use disorders, family history of psychiatric disorders, ongoing medication, and (previous) use of anabolic steroid and/or hormonal supplements. Hormonal contraception use was no exclusion criterion.

The study was approved by the ethical committee of Karolinska Institute (application number: Dnr 2011/281–31/4) and each participant provided signed informed consent according to the declaration of Helsinki before entering the study.

Sexual orientation was assessed using the self-report Kinsey scale^57^, a seven-point scale ranging from 0 (heterosexual, i.e. sexually attracted to the opposite assigned sex at birth) to 6 (homosexual, i.e. sexually attracted to the same assigned sex at birth). Prior to the assessment, TrM and TrW were informed that the Kinsey scale was originally constructed for cisgender individuals and asked to interpret “homosexual” as gynephilic, thus attracted to women/“heterosexual” as androphilic, thus attracted to men, in case the participant was a TrM, and “homosexual” as androphilic/ “heterosexual” as gynephilic when the participant was a TrW.

### Magnetic Resonance Imaging

#### Data acquisition

Magnetic resonance imaging data was acquired on a 3-Tesla MRI scanner (Discovery 3 T GE-MR750, General Electric, Milwaukee, Wisconsin) equipped with a 32-channel/or 8-channel phased array receiving coil. 3D T1-weighted Spoiled Gradient Echo pulse sequence (SPGR) images were acquired with 1 mm^3^ isotropic voxel size (TE = 3.1 ms, TR = 7.9 ms, TI = 450 ms, FoV = 24 cm, 176 axial slices, flip angle 12 deg.). Multi-slice diffusion-weighted imaging was performed using an echo planar imaging sequence with 1 × 1 mm in-plane resolution, [TE = 83 ms, TR 8000 ms, FoV = 24 cm, 60 interleaved axial slices, thickness = 2.9 mm, 60 diffusion gradient directions (b = 1000)]. For the diffusion sequences we used a 32-channel phased array receiving coil, and for the T1 sequence we used an 8-channel coil because it provided better demarcations between white and grey matter in the occipital cortex for the purposes of the Freesurfer analyses (http://freesurfer.net/) used for calculation of ICVs.

#### Data analysis – total intracranial volumes

T1-weighted images were processed using the FreeSurfer image analysis suite, version 5.1^58^ (www.surfer.nmr.mgh.harvard.edu). Volumetric segmentation was performed to derive total ICV measures for each participant. Group comparisons on ICV were subsequently done in SPSS Statistics 21 (SPSS Inc., Chicago, IL). In order to investigate whether transgender (wo)men showed sex-typical or –atypical ICV, we conducted within-sex (assigned at birth) and between-sex group comparisons by means of one-way ANOVA and post-hoc two-sample *t*-tests, considering effects with *p* < 0.05 as significant.

#### Data analysis – diffusion tensor imaging

Diffusion images were analyzed and corrected for (motion) artifacts and eddy current distortions using DTIPrep^59^. Using DTIfit, part of the FMRIB’s Diffusion Toolbox implemented in FSL v5.0 (FMRIB Software Library, Oxford, http://fsl.fmrib.ox.ac.uk/), images were realigned to one of the non-weighted images using affine registration, non-brain tissue removed using BET (part of FSL), and finally a tensor model was fitted to the diffusion data, defining the eigenvalues of the tensor for each voxel to calculate individual FA maps. Voxel-wise statistical analyses were performed using TBSS. Participants’ FA maps were registered to the FMRIB58_FA template, and then transformed to MNI152 space. The normalized individual FA maps were averaged to create a group-wise mean FA white matter skeleton, separately for each group comparison. A threshold of 0.2 was applied to reduce partial volume effects. We tested whole-brain, voxel-wise differences in FA between groups using Randomise (part of FSL), with permutation-based non-parametric testing (5,000 permutations), and applying the Threshold-Free Cluster Enhancement option.

We defined three binary factors: Sex (male or female sex assigned at birth), Sexual orientation (gynephilic or androphilic), and Gender identity (male or female identification). We considered an overall 2 × 2 × 2 (Sex, Sexual orientation, Gender identity) ANOVA not suitable, because the factor Sexual orientation was not binary (homo- or heterosexual) in case of the transgender groups. We therefore decided to answer our research questions: a) whether FA varied as a function of sexual orientation in only the cisgender groups; and b) whether FA varied as a function of Gender identity in all cis- and transgender groups. Furthermore, two one-way ANOVA, followed by post-hoc comparisons were done to specifically test the influence of sex, gender identity, and sexual orientation within groups of the same sex assigned at birth: 1. TrW, HoM, and HeM; 2. TrM, HoW, and HeW, including age as covariate of no interest, respectively. Finally, we investigated sex differences between the six groups of (at birth assigned) males and females by means of one-way ANOVA and post-hoc two sample t-tests. All results, if not specified otherwise, were considered significant at *p*_FWE_ < 0.05 (family-wise error corrected) and a minimal cluster size of *k* > 100 voxel.

In addition to the whole-brain approach, mean FA values of specific tracts (CST, SLF, ILF, IFOF, CC) were extracted that were reported to be sexually dimorphic^28,32–39^. Average FA values per tract were determined for each individual FA map, using AFNI. Anatomical locations and tracts of interest were identified (max. probability threshold of 25, and 1 mm isotropic voxels) using the JHU White Matter Tractography atlas and JHU ICBM-DTI-81 white matter labels^60–62^.

Individual tract-specific FA data were then transferred to SPSS (version 21, SPSS Inc., Chicago, IL) for subsequent group comparisons, with (main manuscript text) and without (Supplementary Results) scores on the Kinsey scale added as covariate of no interest. For exploratory purposes, the transgender groups were subdivided into those with a homosexual orientation (defined as Kinsey scores 4–6) and a non-homosexual (hetero-, or bisexual) orientation (defined as Kinsey ≤3).

## Electronic supplementary material

Supplementary Results
