# Supplementary material for: Structural connections in the brain in relation to gender identity and sexual orientation
Source: Sci Rep. 2017 Dec 20;7:17954. doi: 10.1038/s41598-017-17352-8 (PMC5738422; doi:10.1038/s41598-017-17352-8)
Supplement: Supplementary file 1 — Supplementary Results [file 41598_2017_17352_MOESM1_ESM.doc]

***Structural connections in the brain in relation to gender identity and sexual orientation***

*Sarah M. Burke*^1, 2^, Amir H. Manzouri^3^, Ivanka Savic^2^*

^1^Brain & Development Research Centre, Department of Developmental and Educational Psychology, Leiden University, Leiden, the Netherlands

^2^Department of Women’s and Children’s Health, Karolinska Institute and University Hospital, Stockholm, Sweden

^3^Stressmotagningen, S:t Göransgatan 84, 112 38 Stockholm, Sweden

**SUPPLEMENTARY RESULTS**

**FA – tract-wise group comparisons**

A 2 (Sex) by 2 (Gender identity) multivariate ANOVA, including age, but not Kinsey scores as covariate of no interest, revealed a significant interaction effect in the bilateral IFOF (left: *p* = .024, right: *p* = .003), right SLF (*p* = .016), and forceps major (*p* = .020).

The main effect of Sex was significant for the bilateral CST (left: *p* = .003; right: *p* = .019), and bilateral SLF (left: *p* = .041; right: *p* = .015). Interestingly, the main effect of Gender identity was significant for the bilateral IFOF (left: *p* = .030; right: *p* = .005), and left ILF (*p* = .036) with male-identifying groups (HeM, HoM, TrM) showing higher FA than female-identifying (HeW, HoW, TrW) (Figure 3).

One-way ANOVA including the three male groups (sex assigned at birth) was significant for the bilateral IFOF (left: *p* = .002; right: *p* < .001), bilateral ILF (left: *p* = .013; right: *p* = .024), right SLF (*p* = .023), forceps major (*p* = .044), forceps minor (*p* = .042), and left CST (*p* = .050), revealing that **TrW** had lower mean FA than both cisgender male groups for all these tracts (see Figure 3).

Independent two-sample t-tests showed that also in relation to both cisgender female groups TrW had significantly lower mean FA: compared to HoW in the bilateral IFOF (left: *p* = .019; right: *p* = .016), bilateral ILF (left: *p* = .023; right: *p* = .021), and the splenium of the CC (*p* = .049), and compared to HeW in the left ILF (*p* = .018) and right IFOF (*p* = .033). Thus, TrW had significantly lower FA values compared to all other groups in the IFOF and left ILF, showing a sex-atypical pattern of FA.

In contrast, and similar as for the whole-brain comparisons, one-way ANOVA including the three female (sex assigned at birth) groups revealed no significant differences between **TrM** and HeW or HoW in mean FA per tract. However, visual inspection of Figure 3, in particular the right IFOF, suggests higher FA in both TrM and HoW compared with HeW. Overall, there was a higher standard deviation of FA in TrM than in the other groups, which could explain why the seemingly higher FA values in the IFOF were not significantly different.

One-way ANOVA and subsequent two-group comparisons for investigating sex differences showed that TrM had significantly lower FA compared with HeM bilaterally in the CST (left: *p* = .003; right: *p* = .015), and SLF (left: *p* = .017; right: *p* = .002), but not in the IFOF. In contrast, compared with HoM, TrM displayed significantly lower FA only in the right SLF (*p* = .021) Thus, TrM showed a sex-typical pattern in the CST and SLF, but an “in between” pattern (not different from either cisgender male or female controls) for the IFOF.

Comparisons between the two transgender groups showed significant differences in FA in the left ILF (*p* = .017) with TrM having higher FA than TrW, thus following the ‘sex atypical’ pattern, but no difference in any of the other tracts.

| **Supplementary Table S1** |  |  |  |  |  |
| --- | --- | --- | --- | --- | --- |
|  | cluster size, *k* | max. *t*-value | MNI-coordinates | | |
| Region |  |  | x | y | z |
| *HeM - HeW* |  |  |  |  |  |
| L SLF | 399 | 4.6 | -29 | -8 | 17 |
| R SLF | 361 | 4.6 | 29 | -1 | 21 |
| forceps minor, genu of CC | 327 | 4.5 | -13 | 35 | 4 |
| L CST | 174 | 4.8 | -24 | -13 | 24 |
| L CST | 148 | 5.2 | -18 | -7 | 42 |
| R CST | 124 | 4.4 | 7 | -24 | -24 |
| R ILF | 115 | 4.5 | 37 | -12 | -28 |
| R anterior thalamic radiation | 114 | 4.4 | 7 | -32 | -23 |
| R CST | 111 | 4.1 | 12 | -20 | -2 |
| R anterior thalamic radiation | 103 | 4.4 | 8 | -10 | -14 |
| Whole-brain voxel-wise comparison of a subsample of 30 cisgender heterosexual men (HeM) and 30 cisgender heterosexual women (HeW); results are displayed at *p_FWE_* < .05, cluster threshold of *t* = 2.3, and a minimal cluster size of *k* ≥ 100 voxel; CST = cortico-spinal tract; CC = corpus callosum; SLF = superior longitudinal fasciculus; ILF = inferior longitudinal fasciculus; L = left; R = right | | | | | |
|  |  |  |  |  |  |

| **Supplementary Table S2** |  |  |  |  |  |
| --- | --- | --- | --- | --- | --- |
|  | cluster size, *k* | max. *F*-value | MNI-coordinates | | |
| Region |  |  | x | y | z |
| *Main effect Sex* |  |  |  |  |  |
| R IFOF | 5802 | 30.8 | 35 | -40 | 14 |
| L anterior thalamic radiation | 1435 | 42.3 | -19 | -6 | 17 |
| R splenium of CC | 958 | 31.3 | 14 | -31 | 28 |
| L forceps minor | 558 | 29.6 | -11 | -5 | -10 |
| R posterior cingulum | 318 | 13.0 | 11 | -30 | 33 |
| R SLF | 193 | 11.7 | 39 | -8 | 29 |
| L cerebellar WM | 178 | 26.6 | 18 | -67 | -37 |
| L cerebellar WM | 152 | 28.0 | 14 | -64 | -30 |
| L cerebellar WM | 148 | 19.7 | 15 | -49 | -29 |
| R cerebellar WM | 138 | 18.2 | -15 | -46 | -32 |
| R IFOF, frontal part | 105 | 11.6 | 28 | 14 | 12 |
| Results of a whole-brain, voxel-wise 2 (Sex) by 2 (Gender identity) ANOVA, including all six groups (cisgender homosexual and heterosexual men and women, transgender men and women); results are displayed with *p_FWE_* < .05, cluster threshold of *t* = 2.3, and a minimal cluster size of *k* ≥ 100 voxel; CC = corpus callosum; SLF = superior longitudinal fasciculus; WM = white matter; IFOF = inferior fronto-occipital fasciculus; L = left; R = right; cerebellar clusters are technically regarded as less reliable and are therefore not commented in the main text body. | | | | | |
|  |  |  |  |  |  |
